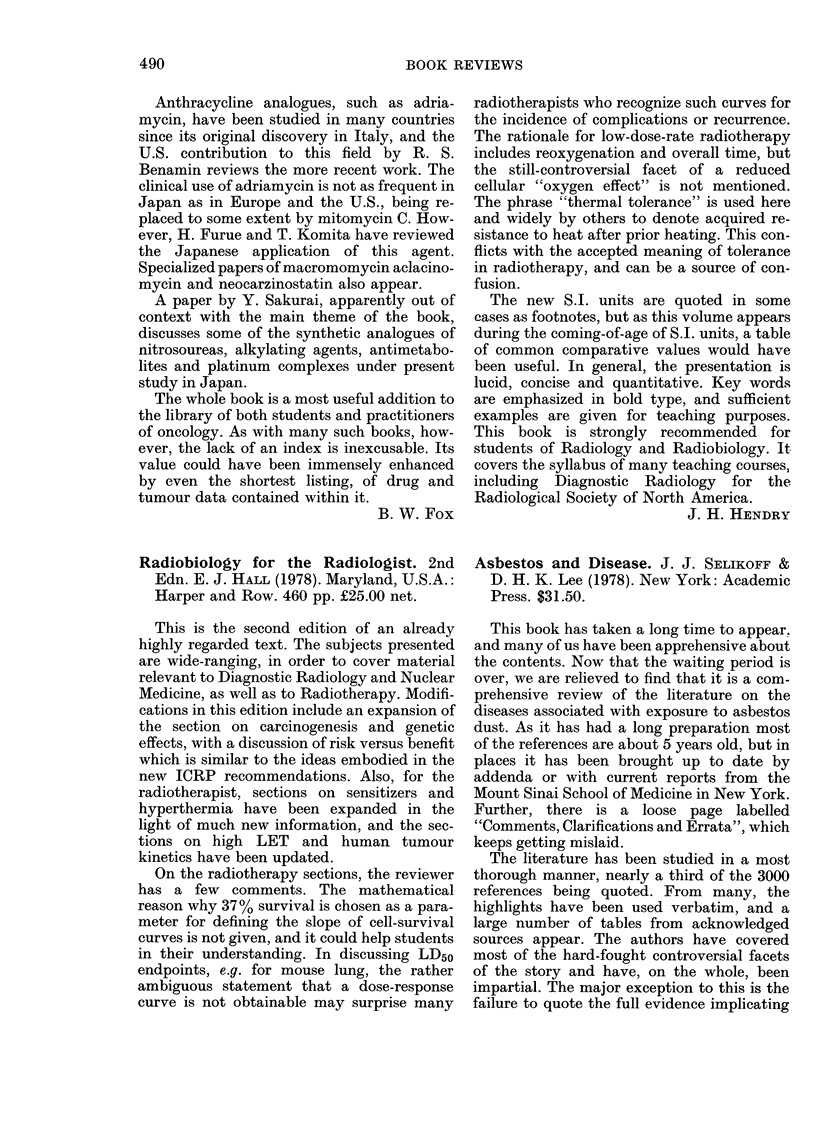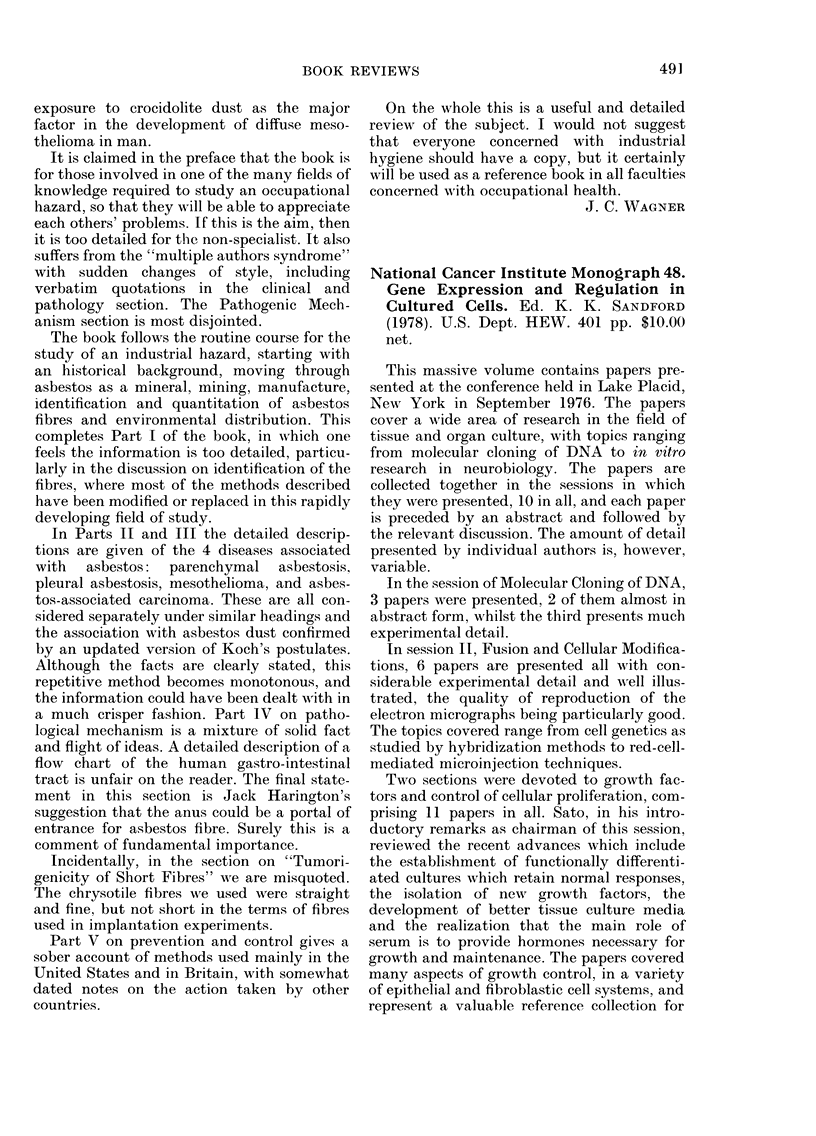# Asbestos and Disease

**Published:** 1979-04

**Authors:** J. C. Wagner


**Asbestos and Disease.** J. J. Selikoff & D. H. K. Lee (1978). New York: Academic Press. $31.50.

This book has taken a long time to appear, and many of us have been apprehensive about the contents. Now that the waiting period is over, we are relieved to find that it is a comprehensive review of the literature on the diseases associated with exposure to asbestos dust. As it has had a long preparation most of the references are about 5 years old, but in places it has been brought up to date by addenda or with current reports from the Mount Sinai School of Medicine in New York. Further, there is a loose page labelled "Comments, Clarifications and Errata", which keeps getting mislaid.

The literature has been studied in a most thorough manner, nearly a third of the 3000 references being quoted. From many, the highlights have been used verbatim, and a large number of tables from acknowledged sources appear. The authors have covered most of the hard-fought controversial facets of the story and have, on the whole, been impartial. The major exception to this is the failure to quote the full evidence implicating exposure to crocidolite dust as the major factor in the development of diffuse mesothelioma in man.

It is claimed in the preface that the book is for those involved in one of the many fields of knowledge required to study an occupational hazard, so that they will be able to appreciate each others' problems. If this is the aim, then it is too detailed for the non-specialist. It also suffers from the "multiple authors syndrome" with sudden changes of style, including verbatim quotations in the clinical and pathology section. The Pathogenic Mechanism section is most disjointed.

The book follows the routine course for the study of an industrial hazard, starting with an historical background, moving through asbestos as a mineral, mining, manufacture, identification and quantitation of asbestos fibres and environmental distribution. This completes Part I of the book, in which one feels the information is too detailed, particularly in the discussion on identification of the fibres, where most of the methods described have been modified or replaced in this rapidly developing field of study.

In Parts II and III the detailed descriptions are given of the 4 diseases associated with asbestos: parenchymal asbestosis, pleural asbestosis, mesothelioma, and asbestos-associated carcinoma. These are all considered separately under similar headings and the association with asbestos dust confirmed by an updated version of Koch's postulates. Although the facts are clearly stated, this repetitive method becomes monotonous, and the information could have been dealt with in a much crisper fashion. Part IV on pathological mechanism is a mixture of solid fact and flight of ideas. A detailed description of a flow chart of the human gastro-intestinal tract is unfair on the reader. The final statement in this section is Jack Harington's suggestion that the anus could be a portal of entrance for asbestos fibre. Surely this is a comment of fundamental importance.

Incidentally, in the section on "Tumorigenicity of Short Fibres" we are misquoted. The chrysotile fibres we used were straight and fine, but not short in the terms of fibres used in implantation experiments.

Part V on prevention and control gives a sober account of methods used mainly in the United States and in Britain, with somewhat dated notes on the action taken by other countries.

On the whole this is a useful and detailed review of the subject. I would not suggest that everyone concerned with industrial hygiene should have a copy, but it certainly will be used as a reference book in all faculties concerned with occupational health.

J. C. WAGNER